# Tailoring remote patient management in cardiovascular risk management for healthcare professionals using panel management: a qualitative study

**DOI:** 10.1186/s12875-024-02355-y

**Published:** 2024-04-20

**Authors:** Margot Rakers, Nicoline van Hattem, Iris Simic, Niels Chavannes, Petra van Peet, Tobias Bonten, Rimke Vos, Hendrikus van Os

**Affiliations:** 1 Department of Public Health and Primary Care, Leiden University Medical Centre, Leiden, 2333 ZA The Netherlands; 2 Health Campus the Hague, Leiden University Medical Center, The Hague, 2511 DP The Netherlands

**Keywords:** Remote patient management, Panel management, Proactive care, Prevention and management, Cardiovascular disease

## Abstract

**Background:**

While remote patient management (RPM) has the potential to assist in achieving treatment targets for cardiovascular risk factors in primary care, its effectiveness may vary among different patient subgroups. Panel management, which involves proactive care for specific patient risk groups, could offer a promising approach to tailor RPM to these groups. This study aims to (i) assess the perception of healthcare professionals and other stakeholders regarding the adoption and (ii) identify the barriers and facilitators for successfully implementing such a panel management approach.

**Methods:**

In total, nineteen semi-structured interviews and two focus groups were conducted in the Netherlands. Three authors reviewed the audited transcripts. The Consolidated Framework for Implementation Strategies (CFIR) domains were used for the thematic analysis.

**Results:**

A total of 24 participants (GPs, nurses, health insurers, project managers, and IT consultants) participated. Overall, a panel management approach to RPM in primary care was considered valuable by various stakeholders. Implementation barriers encompassed concerns about missing necessary risk factors for patient stratification, additional clinical and technical tasks for nurses, and reimbursement agreements. Facilitators included tailoring consultation frequency and early detection of at-risk patients, an implementation manager accountable for supervising project procedures and establishing agreements on assessing implementation metrics, and ambassador roles.

**Conclusion:**

Panel management could enhance proactive care and accurately identify which patients could benefit most from RPM to mitigate CVD risk. For successful implementation, we recommend having clear agreements on technical support, financial infrastructure and the criteria for measuring evaluation outcomes.

**Supplementary Information:**

The online version contains supplementary material available at 10.1186/s12875-024-02355-y.

## Introduction

Cardiovascular diseases (CVD) are among the leading causes of death globally, resulting in an increasing disease burden and associated costs [1, 2]. Treating modifiable risk factors for CVD, such as lowering elevated blood pressure and losing weight, is imperative [3, 4]. However, controlling risk factors in clinical practice still has considerable room for improvement [5, 6]. Providing in-person care by a healthcare professional combined with remote patient management (RPM) interventions, a digital health platform that facilitates the assessment of patients outside their usual clinical setting [7], has proven effective in many cases [8–12]. For instance, utilising digital wearables with online feedback has been successful in helping patients with uncontrolled hypertension achieve their blood pressure goals and improve their lifestyles [13]. To ensure its success, an RPM implementation strategy must be designed to meet all end-user needs. However, this can be particularly challenging in cardiovascular risk management (CVRM), given numerous patient subgroups with varying health or social issues, resulting in suboptimal control of cardiovascular risk factors [14–16]. These subgroups, also referred to as panels, can be identified using routine care data from electronic medical records (EMR) [17]. This proactive approach, known as panel management, enables healthcare professionals to systematically allocate appropriate interventions that are tailored to the clinical and social needs of a specific patient panel [18–22]. Therefore, panel management could be a promising approach to tailor RPM interventions.

Currently, an RPM intervention is being developed by the public-private Connect@Heart consortium in Leiden region, The Netherlands, to support healthcare professionals (HCPs) in tailoring the use of RPM in CVRM. To increase the uptake of this intervention, involving all end-users in an early stage of development is crucial to achieving successful implementation and preventing low clinical adoption [23–28]. Despite the potential promises of panel management, few studies have been conducted to explore the perceptions of HCPs involved in cardiovascular panel management programmes using RPM. Hence, this qualitative study aimed (i) to assess the perception of HCPs and other key stakeholders of the adoption of a panel management approach to tailor an RPM intervention to specific patient risk groups and (ii) to identify the barriers and facilitators for successful implementation of a panel management approach for RPM for cardiovascular risk factor control in primary care.

## Methods

### Study design

**Fig. 1:**
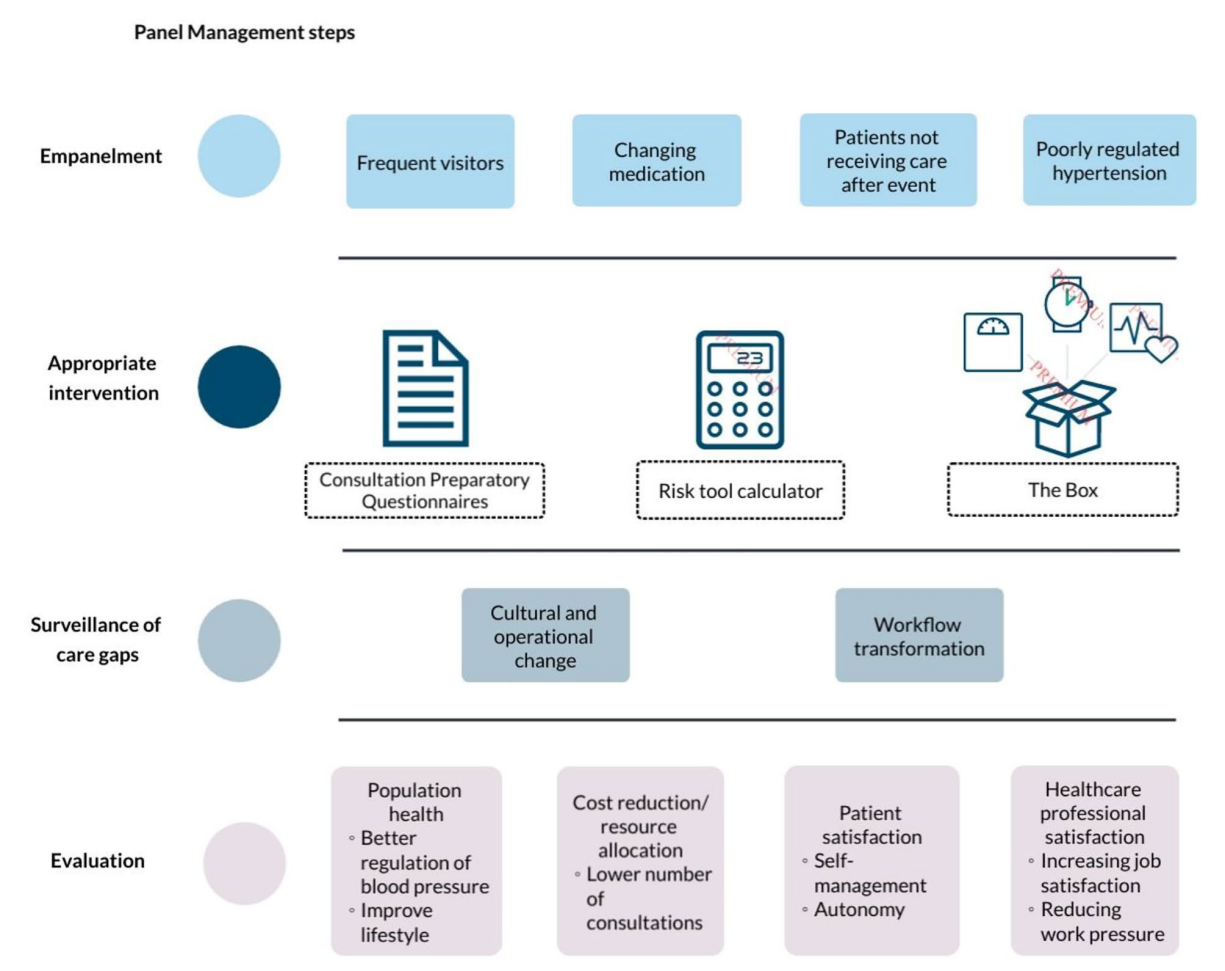
Panel Management steps in this study The Box: Our RPM intervention comprises a blood pressure monitor, scale, and activity meter for home monitoring. Consultation Preparatory Questionnaires: Digital questionnaires with standard questions related to cardiovascular checks

The Connect@Heart consortium aims to collaboratively create, implement, and evaluate an RPM intervention for controlling cardiovascular risk factors and improving lifestyles in primary care. The aim is to enhance the implementation of an RPM intervention by utilising a panel management approach (see Fig. 1). The RPM intervention comprises a validated RPM infrastructure containing a blood pressure monitor, a weigh scale, and an activity tracker [29], along with digital questionnaires for consultative preparations. These digital questionnaires, which include cardiovascular assessment questions, are sent to patients prior to their appointments. ‘The RPM infrastructure is designed to support the management of CVRM patients in their clinical environment and to increase their health awareness,. It is fully integrated into the EMR system. Furthermore, in the Netherlands, RPM is not yet standardised on a national level. However, it has seen increased utilisation in the past years, for various health indications such as COPD, COVID-19, and diabetes [30–32]. As of the time of writing, hospital care has had a reimbursement provision for telemonitoring integrated into the regular funding system for the past year. However, there is no structural reimbursement for using RPM in primary care [33].’

Panel management consists of four steps: (i) the identification of people sharing the same risk of an adverse event in care and allocating them to an administrative subgroup (panel), (ii) the allocation of the appropriate intervention to each panel, (iii) the identification of those having missed the appropriate intervention at the chosen time, and (iv) the evaluation of the panel management programme [18–22]. The current qualitative study is an initial assessment of this panel management approach with the RPM intervention and focuses on the first, second, and third step of panel management, while the fourth step will be evaluated in a real-world context in the following study stage. Fig. 1 illustrates the panel management steps, and an extensive explanation of our panel management approach can be found in Supplementary Fig. S1.

Data were reported following the Standards for Reporting Qualitative Research (SRQR) [34]. The study was conducted according to the guidelines in the Declaration of Helsinki, and all procedures involving research study participants were approved by the Ethics Committee of the Leiden University Medical Center (N21.126).

### Participants and recruitment

Interviews were conducted with Dutch-speaking general practitioners (GPs) and practice nurses (PNs). To account for variability, GPs and PNs from different practices were approached through the snowball and purposive sampling technique to ensure a balanced distribution of sex, age, and work experience. Sampling was stopped when no new information emerged, and data saturation was reached. GPs, PNs, and other crucial stakeholders (health insurers, project managers, and IT consultants) were purposefully selected for the focus group discussions. GPs and PNs who took part in the focus groups adhered to the same criteria used for the interviews. Additional essential stakeholders were chosen based on their familiarity with comparable RPM infrastructures in the Leiden region. Participants expressing interest were invited via email or telephone to schedule an appointment. Recruitment occurred between December 2021 and April 2022.

### Data collection

All interviews and focus group discussions were conducted online using Microsoft Teams, with both video and audio recording. Informed consent was reconfirmed and recorded at the start of each interview and focus group. The interviews were conducted between January 2022 and July 2022 by a single researcher (MR) who received training in qualitative research from an expert (PP). After the interviews, two multi-disciplinary focus group discussions were held with GPs, PNs, and other stakeholders to facilitate open discussions and provide more comprehensive insights into the panel management approach and its implementation from various stakeholder perspectives. During the focus groups, NvH provided support to the first author. Interviews typically lasted 30–60 min, while each focus group lasted 100–120 min.

Topic lists guided the semi-structured interview and focus group discussions based on the Consolidated Framework for Implementation Research (CFIR) and expert opinion (Supplementary Text S2). The CFIR framework is a combination of multiple implementation theories that can be applied to facilitate the design, evaluation, and implementation of interventions. The CFIR framework entails 39 constructs within five domains: *Intervention characteristics* (the three panel management steps (1), empanel management, (2), ppropriate interventions, and (3) evaluation of the panel management approach), *Outer setting* (the context in which healthcare professionals are situated), *Inner Setting* (healthcare professionals workflows and involved stakeholders), *Characteristics of the individual* (the healthcare professionals and involved stakeholders) and *Process* (used for implementation of the panel management approach) [35]. Before the interviews and focus groups, a handout was sent to the participants to provide them with information about the aim, the design, and the content of the panel management approach (see Supplementary Fig. S1). The interview protocol included open-ended questions centered on the following subjects: (a) participants’ perspective on current cardiovascular risk management, (b) the value of a panel management approach for successfully identifying patient risk groups, (c) the appropriate RPM intervention, and (d) barriers and facilitators for implementation of each of the panel management steps.

Three authors (MR, IS, and NvH) transcribed the interviews verbatim using Microsoft Word, pseudonymised them, and subsequently cross-checked them for discrepancies against the original recordings. A lay summary was made available to participants upon request.

### Data analysis

Using Atlas.ti software (version 8.4), qualitative data obtained from the semi-structured interviews were analysed using the Framework Method following a deductive approach based on the five steps outlined by Ritchie and Spencer: (1) familiarisation, (2) identifying a framework (the research team selected the CFIR as the a priori framework), (3) indexing subsequent transcripts using the existing constructs and domains, (4) charting by summarising data from each transcript, and (5) interpreting the data [36]. Three authors (MR, IS, and NvH) reviewed the initial coding to ensure consistency and establish a shared understanding of the CFIR domains and constructs that had been identified. Inductive coding was utilised to formulate novel codes in cases where portions of the transcripts did not encompass information within the existing CFIR constructs. This encompassed capturing perspectives on panel management adoption (first research aim) and defining panels (Supplementary Table S1). This process confirmed the definition of the codes and ensured their accurate representation in the research (see Supplementary Table S5). Quotes that best encapsulated the perspectives and findings for each construct were selected. The constructs with the most substantial content were chosen to provide a comprehensive description. To provide context for the analysis, data extracts were identified by participant number, self-disclosed sex, and age to offer contextual information.

### Results

#### Characteristics

Table 1 overviews the 19 HCPs and five other stakeholders who participated in this qualitative study. The interviews included 9 GPs and 10 PNs from 10 primary care practice centres, with a mean age of 41 and a range of 31–65 years. The focus group discussions included three GPs, three PNs, two project managers, two IT-domain experts and one health insurer. The three GPs and three PNs were also involved in the interviews.

**Table 1:** Overview of the participants

Participants interviews								
Professional ID	Profession - specialty	Gender (Male/ female)	Age group	Seniority	Years of experience with RPM	Practice size (rounded)	Patient population	
1	GP – musculoskeletal	Female	40–50	> 10 years	5–10 years	3310	Highly educated, elderly	
2	GP – elderly	Female	40–50	> 10 years	5 years	4450	Highly/low educated, elderly, young	
3	GP – educator	Male	30–40	5–10 years	< 5 years	2920	Average population (reflecting NL population)	
4	GP – educator/ICT/HR	Male	40–50	> 10 years	5–10 years	2720	Average population (reflecting NL population)	
5	PN – somatics/ practice manager	Female	40–50	> 10 years	5–10 years	2870	Elderly, average population (reflecting NL population)	
6	GP – cardiovascular	Male	60–70	> 10 years	5–10 years	2600	Low-educated, immigrants	
7	PN – somatics	Female	30–40	< 5 years	< 5 years	3390	Average population (reflecting NL population)	
8	PN – elderly	Female	60–70	> 10 years	10 years	3890	Average population (reflecting NL population)	
9	GP - diabetes	Female	30–40	< 5 years	< 5 years	2500	Low-educated	
10	PN – somatics	Female	40–50	> 10 years	10 years	2580	Low-educated/immigrants	
11	GP – elderly	Male	60–70	> 10 years	5 years	2230	Average population (reflecting NL population)	
12	PN – somatics	Female	30–40	5–10 years	< 5 years	2200	Elderly, average population (reflecting NL population)	
13	PN – elderly	Female	30–40	5–10 years	< 5 years	3010	Average population (reflecting NL population)	
14	GP – educator	Male	40–50	> 10 years	< 5 years	3010	Average population (reflecting NL population)	
15	GP - policy and management	Female	40–50	> 10 years	5–10 years	3200	Average population (reflecting NL population)	
16	PN – elderly	Female	50–60	> 10 years	< 5 years	2600	Low-educated, elderly	
17	PN – somatics	Female	50–60	> 10 years	5–10 years	2300	High-educated	
18	GP – educator	Female	30–40	5–10 years	< 5 years	3900	High-educated, average population	
19	PN – somatics	Female	50–60	> 10 years	< 5 years	3200	Average population (reflecting NL population)	
**Participants focus groups**								
20	Project manager – eHealth and social domain	Male	50–60	> 10 years	5 years	-	-	
21	Project manager - eHealth	Female	30–40	> 10 years	5–10 years	-	-	
22	Health insurer – primary care and digitisation	Male	60–70	> 10 years	5 years	-	-	
23	IT-consultant – development	Male	50–60	> 10 years	5 years	-	-	
24	IT consultant – data infrastructure	Male	50–60	> 10 years	10–15 years	-	-	
3	GP – educator	Male	30–40	5–10 years	5–10 years	2920	Average population (reflecting NL population)	
7	PN – somatics	Female	30–40	< 5 years	< 5 years	3390	Average population (reflecting NL population)	
9	GP – diabetes	Female	30–40	< 5 years	< 5 years	2500	Low-educated	
10	PN – somatics	Female	40–50	> 10 years	10 years	2580	Low-educated/immigrants	
12	PN – somatics	Female	30–40	5–10 years	< 5years	2200	Elderly, average population (reflecting NL population)	
14	GP – educator	Male	40–50	> 10 years	< 5 years	3010	Average population (reflecting NL population)	

GP: general practitioner; PN: practice nurse; ICT: information and communication technology; HR: human resources

### The adoption of a panel management approach

Most participants experienced the current CVRM workflow functioning sufficiently well. Nevertheless, they emphasised the potential for enhancing the personalisation of care for patients with varying levels of CVD risk (see Supplementary Table S1). ‘*In itself it does work, but I am concerned about the frequency of visits of patients. It may not be necessary for them to come in so often, and at times it might be more beneficial to have a new person scheduled for that time slot instead.’ [Professional ID: 13]* Participants also mentioned the challenge of identifying everyone at risk of CVD. They perceived the current CVRM approach as mainly reactive rather than proactive, underscoring the importance of tailored healthcare. *‘There is a significant demand for customised interventions, incorporating individualised adjustments, which could be effectively achieved by implementing intelligent algorithms.’ [Professional ID: 4].*Participants argued that panel management had the potential to enhance personalised care and proactive health policies.

### Identification of potential barriers and facilitators

**Fig. 2:**
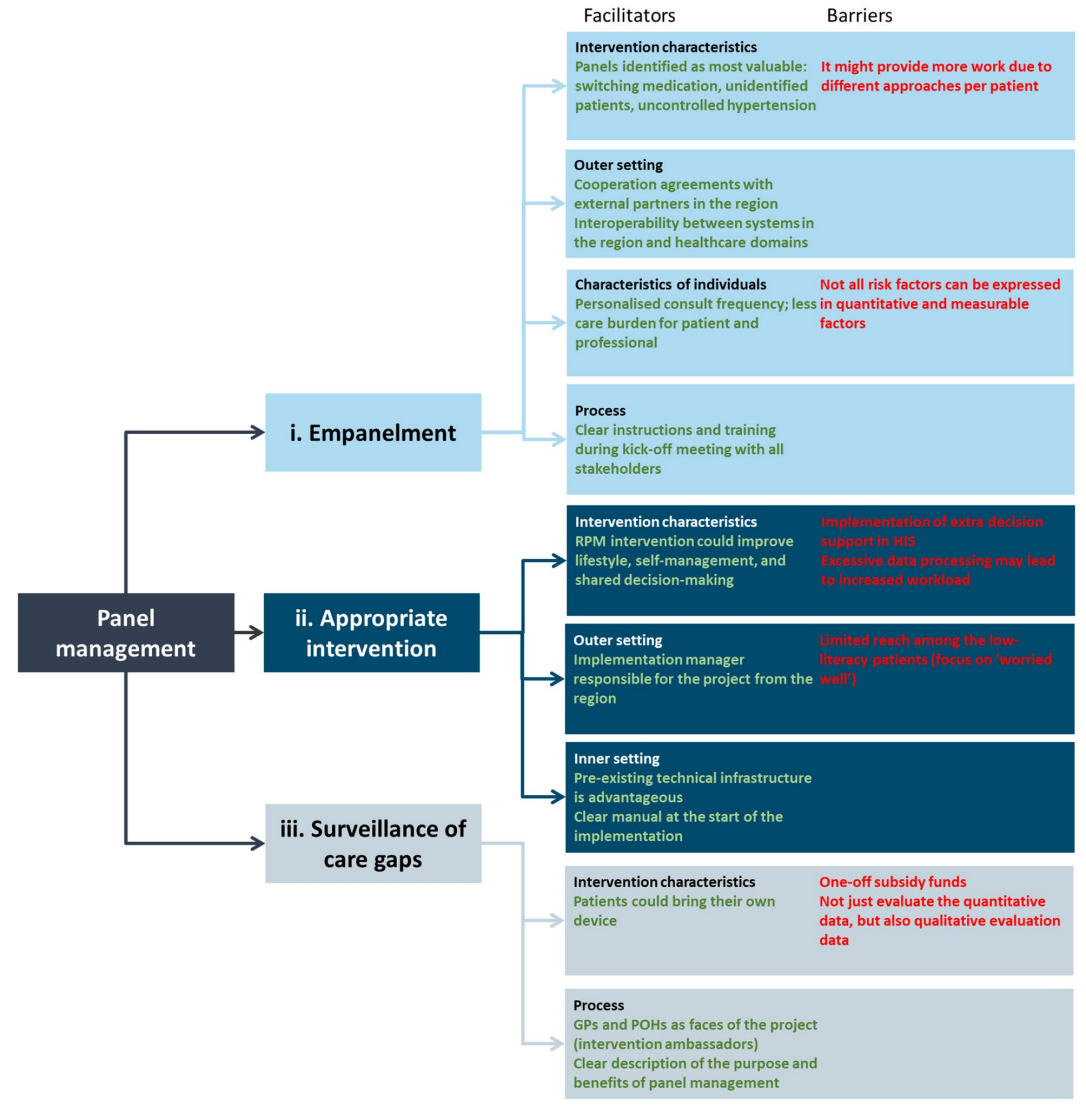
The most important barriers and facilitators for successful implementation per step in this panel management approach for RPM for cardiovascular risk factor control in primary care

Regarding the prespecified CFIR domains, the most important barriers and facilitators of each panel management step were summarised in Fig. 2. An extensive overview of all the barriers and facilitators can be found in Supplementary Tables S2, S3, and S4.

#### Panel management steps

#### Step i. Empanelment

##### Intervention characteristics

Different perspectives were expressed regarding identifying individuals with a similar risk of adverse care events and their allocation to administrative subgroups. GPs suggested that patients with well-controlled blood pressure or those at low risk of complications would benefit the most from RPM. According to them, such patients did not necessarily need in-person visits but could be monitored remotely. GPs also pointed out that patients starting or changing medications might require a significant number of consultations in a short period, making RPM beneficial for them as well. In contrast, PNs recommended including individuals with unhealthy lifestyle habits, comorbidities (such as diabetes and poor kidney function), and young adults at high risk in the RPM panel. Four participants expressed the view that digital health should not be limited to panels but should be widely accessible and customisable based on individual needs rather than solely their level of risk. Additionally, some professionals suggested that stratifying patients into groups and linking them to different interventions might distribute their workload more effectively. *‘Indeed, it generates additional work as different approaches are required for each group, thereby necessitating filtering and subsequent actions to be taken.’ [Participant ID: 3]*.

##### Outer setting

All stakeholders considered collaboration with external partners essential for establishing the digital infrastructure. ‘*The implementation needs to occur via the care groups (general practices that are responsible for coordinating and delivering care) within a unified regional organisation, in which collaborative plans align with identical quality policies (…) and the advantage is that we operate using the same EHR system, all managed uniformly.’ [Participant ID: 6]* Primary care practices in the region had formed close partnerships with collaborators responsible for the technical infrastructure, which was crucial for implementing the panel management approach alongside the RPM intervention. This collaboration will additionally simplify the utilisation of risk stratification tools, which leverage risk algorithms developed by private technology developers.

##### Characteristics of individuals

Most participants valued an empanelment approach, leading to a more tailored consultation frequency and potentially reducing the time needed, thus lessening the burden on patients and professionals. However, some individuals expressed concerns about the effectiveness of this empanelment approach, stating that not all risk factors necessary for stratification could be accurately captured through quantifiable metrics alone. ‘*It is not always easy to get a complete picture of everyone’s situation due to incomplete registration of ICPC (International Classification of Primary Care) codes in our EHR.’ [Participant ID: 1]* Factors like social determinants, including socioeconomic status, language, and literacy skills, might not be fully reflected in the available routine data and, therefore, might not always be recorded in the EMR.

##### Process

Utilising risk algorithms to stratify the primary care practice population into patient panels is a relatively new field and operating method. Consequently, participants advised that clear communication with all stakeholders engaged in the implementation regarding the purpose and advantages of the risk stratification approach is vital for its adoption. Practical methods such as training during kick-off meetings and information sessions at the primary care practice centers were identified as effective ways to promote this communication. *‘For effective engagement, it is essential for individuals to be well-informed about the process and benefits of stratification. Therefore, the system should be designed to be easy to use, requiring minimal time and effort to navigate, and approachable to enhance user adoption.’ [Participant ID: 18]*.

#### Step ii. Appropriate intervention

##### Intervention characteristics

GPs and PNs suggested that the proposed RPM intervention could enhance lifestyle, self-management, and shared decision-making, especially when considering reimbursement, education, and technical support. Furthermore, the RPM intervention was anticipated to provide a population-level perspective without incurring direct labor costs. ‘*By enabling the reallocation of time spent on low-risk patients, digital health technologies can potentially improve care for high-risk patients. Thus, where feasible, removing this burden of low-risk patients represents a vital aspect of such technologies*.’ *[Participant ID: 6 & 24]* Anticipated adverse outcomes included excessive data to be processed from home measurements, which could burden PNs due to extra data processing, additional questions and extra consultations, whether in-person or over the phone. Additional potential obstacles to adoption included introducing a supplementary digital dashboard alongside the current EMR and the inability to use devices that patients had purchased independently.

##### Outer setting

Despite the potential of the interventions to improve self-management skills among patients, concerns were raised about their limited effectiveness among patients with low literacy levels, as these interventions might primarily benefit the ‘worried well’. *‘I find it challenging. The group that is already motivated for lifestyle changes is usually easier to engage with the Box because they are often already actively engaged in a healthier lifestyle. For example, they already have access to their lab results through the online patient portal and report their weight and blood pressure.’ [Participant ID: 11]* Participants emphasised the significance of collaborating with patient organisations. Regarding the implementation process, HCPs and project managers stressed the importance of having an implementation and/or project manager (distinct from the PN or practice manager) responsible for overseeing the project’s processes and coordinating with external partners.

##### Inner setting

When asked about integrating interventions into existing workflows, GPs and PNs indicated that having a pre-existing technical infrastructure (including dataflow and integration of wearables) in partnership with the private entities responsible for that infrastructure is beneficial. To improve the integration of workflows in a new practice, a project manager recommended providing a clear and universally understandable manual at the beginning of implementation. *‘There should be a clear protocol for managing patient panels. (…) There should also be a standardised implementation plan that every practice can use uniformly.’ [Participant ID: 14]*.

#### Step iii. Surveillance of care gaps

##### Intervention characteristics

The participants, especially GPs and PNs, extensively discussed the costs associated with the intervention and the responsibility for financing it. They were aware that funding presented a significant barrier to implementing such interventions, primarily due to reliance on one-time subsidies rather than structural reimbursement and the lack of compensation for the additional time required for end-users to implement the intervention. A healthcare insurance provider emphasised that the focus should not only be on cost reduction but also prioritise the efficient allocation of resources for patient care and promote patient and healthcare professional satisfaction. HCPs acknowledged the importance of qualitative data, including patient and professional satisfaction, in addition to clinical outcomes and cost-effectiveness. Furthermore, GPs and PNs revealed that patients often purchase their own blood pressure monitors, especially if they can connect the device to the EPD. “*It would be motivating for patients to receive reimbursement for a trial period, and if they find the device effective, they can then pay a portion of the device cost*,” suggested a GP [*Participant ID: 9].*

##### Process

The significance of intervention ambassadors was highlighted to ensure the active involvement of patients and HCPs. These ambassadors, who can be GPs or PNs, serve as essential opinion leaders and can significantly influence patient attendance at information sessions and compliance with the interventions. *‘Earlier, we organised two evenings attended by 480 people (…) where it was important to have our own GPs present because it turns out that patients are then more likely to participate in the intervention.’ [Participant ID: 3]* Furthermore, it is crucial to clearly explain the purpose and benefits of the panel management approach during contextual activities such as introductions, training sessions, and e-learning courses. Additionally, providing a clear protocol for managing specific patient panels and specifying whom to contact for technical support is essential.

## Discussion

### Summary

In this study, GPs, PNs, health insurers, project managers, and IT consultants perceived panel management as potentially valuable for tailoring an RPM intervention to the needs of patient subgroups. Participants agreed that panel management could initially help identify subgroups at risk of CVD using routine care data from EMR. The main implementation barriers of a panel management approach encompassed (i) concerns about not capturing all risk factors within the EMR necessary for stratification, (ii) additional clinical and technical tasks directed to PNs, and (iii) reimbursement streams of the different components within the panel management approach. The main facilitators included (i) decreasing the care burden for patients and HCPs through tailored consultation frequency and early detection of patients at high-risk, (ii) an implementation manager accountable for supervising projects’ procedures and coordinating with external partners responsible for a pre-existing technical infrastructure, and (iii) clear agreements about the evaluation of implementation indicators and ambassadorship.

### Comparison with existing literature

HCPs in our study believed that the productivity of panel tracking through risk stratification in the EMR depended on integration with the EMR [CFIR: intervention characteristics]. Other studies reported similar results; for example, successful panel management interventions required clinical decision support systems that issue relevant care reminders within the same system the healthcare provider works with [19, 37]. Electronic panel support systems with these features were associated with better chronic disease management, but despite the integration of a panel management support tool, barriers to their use were still encountered. Barriers, such as insufficient time to reach vulnerable patients, technical difficulties, and incomplete data, were also identified, as were our participants [38, 39]. Previous work with HCPs suggested that the appointment of organisational leaders and managers [CFIR: inner setting], along with clear communication and defined team roles, could help overcome the identified barriers, which aligns with the recommendations of our study to enhance the chances of successful adoption [38].

Implementing panel management strategies to enhance CVRM is not a novel concept. Several studies have demonstrated the efficacy of this approach in improving outcomes for patients at high risk for CVD. Most studies utilising panel management techniques to enhance CVRM concentrate on medical factors and healthcare accessibility [40–43]. However, there has been less emphasis on using empanelment to customise digital health interventions for patient panels in CVRM. Additionally, these studies employ panel management assistants or community health workers to provide comprehensive team, administrative, and project support to ensure the efficient management of accounts and successful implementation of the approach [19], assuming that non-clinical staff are also needed to implement such an approach effectively. This aligns with our findings and emphasises the importance of a project manager or panel manager, preferably an external individual rather than a practice manager from the general practice where the RPM is implemented. This individual oversees organisational processes and ensures efficient management. By working across multiple practices, they can offer complementary organisational perspectives. Furthermore, not all healthcare practices are eligible for such implementation, as it depends on their level of digital readiness - i.e., their motivation and competence to adopt, use, and disseminate digital healthcare technologies effectively - for employing RPM. Previous research has reported that perceived competence, rather than motivation, impacts digital readiness [CFIR: process], highlighting the significance of training and education for HCPs and patients [44].

Finally, it is essential to note that while RPM has been suggested as a potential solution to the growing shortage of HCPs, technology as a substitute for in-person consultations may not be equally accessible to all patients. The successful adoption of new digital interventions may depend on factors such as the patients’ age, level of education, interests, physical abilities, familiarity with technology, and availability of support to assist with self-care and functional independence [14]. This echoes the concern of several of our participants that not all risk factors necessary for stratification could be accurately captured through quantifiable factors but also encompass social determinants. It is thus additionally crucial to take into account patients’ preferences for specific interventions independent of their CVD risk level. Different options for appropriate care should continue to be explored accordingly [45]. We acknowledge that the RPM intervention alone is insufficient, and an RPM infrastructure should must also address self-management for lifestyle factors that extend beyond self-measurement. These programs, such as combined lifestyle interventions for weight loss, efficiently provide comprehensive information on maintaining a balanced lifestyle [46]. Previous research has shown that patients who used such platforms felt empowered in their interactions with healthcare providers and well-informed about their condition [47].

### Strengths and limitations

To the best of our knowledge, this is the first qualitative study that investigates the perspectives of HCPs and other key stakeholders regarding implementing RPM through a panel management approach. While RPM implementation has been well-documented, this study uniquely explores an implementation strategy that assigns patient risk groups to an RPM intervention. The study has yielded valuable insights that can be applied to enhance the implementation strategy in future studies, allowing for the effective addressing of identified barriers. Another important strength of this study is its targeted approach, which encompasses not only HCPs but also other critical stakeholders, including IT domain experts, project managers, and healthcare insurers. These stakeholders play pivotal roles in shaping the technical, structural, and financial support required to successfully adopt a given intervention. Additionally, using an established and well-recognised framework, such as the Consolidated Framework for Implementation Research (CFIR), is essential in the field of implementation science. It enables a more comprehensive understanding, a detailed depiction, and an accurate identification of the factors associated with the implementation process.

Our qualitative study also has several limitations. Initially, the snowball sampling method utilised to recruit participants may result in selection bias, as those who exhibit greater involvement with technical innovations are more likely to participate than their counterparts in other primary care practices. Through purposeful sampling, we aimed to include a diverse range of general practices, which encompasses those affiliated with less technical aspects. Second, our qualitative study focused solely on HCPs and other stakeholders, and we did not investigate patients’ views on panel management. We deliberately chose this first phase without patients to identify barriers and facilitators for working proactively to match our implementation strategy early. In the next phase, healthcare providers can introduce the intervention to patients and assess their receptivity in these later stages. Third, data collection in qualitative research is subject to variation due to differences in communication during interviews. Therefore, we employed a pre-determined interview topic list based on CFIR in this study to standardise the process as much as possible and continued interviewing until data saturation was attained.

### Implications for practice and research

GPs and PNs suggested that patients with well-controlled blood pressure, low-risk individuals, and high-risk young adults have the potential to benefit from RPM. Instead of applying RPM to all patients within CVRM universally, there is potential in selectively targeting specific patient risk groups that would benefit the most from RPM. This focused approach holds promise in reducing the need for frequent in-person visits [41]. By doing so, this enables the optimisation of resource allocation, including HCP and patient time, as well as overall costs, all while ensuring that the quality of care and the satisfaction of healthcare providers and patients remain uncompromised.

Given the limitation of accurately recording all essential risk factors, including social determinants of health, within routine care data from EMR, HCPs and researchers should acknowledge the importance of incorporating a broader range of factors in both research and continuous monitoring of the effects of the RPM intervention in practice. This may entail using qualitative assessments, patient-reported data, and additional data sources (e.g., socioeconomic data) to assess outcomes. By embracing a comprehensive approach that includes both quantitative and qualitative measures, a more nuanced understanding of patient risk profiles and the effectiveness of interventions can be achieved.

The results of this study could provide valuable insights for developing an RPM infrastructure that incorporates a panel management approach. The qualitative assessment of this program will eventually serve as the basis for a mixed-method feasibility study, which seeks to determine the viability of providing proactive care to patients at high risk of CVD by effectively allocating resources to the identified patient panels in this investigation.

### Electronic supplementary material

Below is the link to the electronic supplementary material.

Supplementary Material 1

## Data Availability

The data used to support the findings of this study are included within the article. Raw data analysed during the current study are not publicly available due to confidentiality agreements but are available from the corresponding author on reasonable request.

## Abbreviations

RPM: Remote Patient Management
CFIR: Consolidated Framework for Implementation Strategies
CVD: Cardiovascular diseases
CVRM: Cardiovascular risk management
SRQR: Standards for Reporting Qualitative Research
GPs: General practitioners
PNs: Practice nurses
EMR: Electronic medical record
HCPs: Healthcare Professionals
