# Supplementary material for: Tailoring remote patient management in cardiovascular risk management for healthcare professionals using panel management: a qualitative study
Source: BMC Prim Care. 2024 Apr 20;25:122. doi: 10.1186/s12875-024-02355-y (PMC11031879; doi:10.1186/s12875-024-02355-y)
Supplement: Supplementary file 1 — Supplementary Material 1 [file 12875_2024_2355_MOESM1_ESM.docx]

**Table S1 Potential barriers and facilitators for implementation of a panel management approach for cardiovascular disease management – adoption of a panel management approach**

| **Domain and Construct** | **Potential Barriers** | **Potential Facilitators** |
| --- | --- | --- |
| **Current Practice** | | |
| Workflow | Current workflow mainly reactive rather than proactive. | Care burden split between GPs, practice nurses and assistants |
| Advantage |  | Panel management could be enhance personalised care and proactive measures. |
| Disadvantage | Not having an overview of all patients at risk and non-personalised care |  |
|  | No standardised care for low-care patients |  |

**Table S2 Potential barriers and facilitators for implementation of a panel management approach for cardiovascular disease management – empanelment**

|  | **Empanelment** | |
| --- | --- | --- |
| **Domain and Construct** | **Potential Barriers** | **Potential Facilitators** |
| **Intervention characteristics** | | |
| Evidence strength & quality |  | Large amount of structured data |
| Relative advantage |  | Low-risk patient targeting (low-hanging fruit); fewer checks required and more attention for those who need/want their checks physically |
|  |  | Personalizing care delivery (tailored care and identification) |
| Disadvantage | Digitalization is causing us to lose human contact. |  |
| Adaptability | Shift of patient risks and therefore need for constant monitoring of risk factors. |  |
| Design quality and packaging | To many pop-ups/alarm fatigue |  |
|  | Stratifying groups give different linked interventions, so more work. |  |
| Costs |  | Earlier identification has long-term cost benefits |
| Responsibility | Losing track of patients not included in one of the panels |  |
| Requirements |  | Fully integrated into health information system, no endless clicking, no multiple programs. |
| Outcomes |  |  |
|  |  |  |
|  |  |  |
| Panel identification | Panels identified as less valuable: frequent visitors (often need multiple interventions and no digital care), low (health & technical) literacy | Panels identified as most valuable: switching medication, patients between two stools and uncontrolled hypertension |
|  | Panels identified as missing: controlled hypertension, low-risk patients, young patients with high risk, diabetes, poor renal function |  |
| **Outer setting** | | |
| Patient needs & resources | Focus on the 'worried well'; excluding patients who are less digitally proficient | It makes patients aware of what is going on with their health by approaching them proactively |
|  |  | More and more people, including the elderly, are digitally literate |
| Cosmopolitanism |  | Cooperation agreements in the region to install the stratification tool |
| **Inner setting** | | |
| Available resources |  | Interoperability between systems in the region and healthcare domains |
| Formally appointed internal implementation leaders |  | Implementation manager physically visits practices to explain decision rules |
| **Characteristics of individuals** | | |
| Knowledge & beliefs about the intervention | Not all risk factors can be expressed in quantitative and measurable factors | Personalized consult frequency; less care burden for patient and professional |
| Self-efficacy |  | Willingness of professionals to include approach in current workflows |
| Individual stage of change |  | Certain groups (e.g. the elderly) who do not want to change |
| **Process** | | |
| Engaging |  | Provide insight into the purpose and potential benefits of using decision rules |
|  |  | Clear instructions and training during the kick-off meeting with all stakeholders |

**Table S3 Potential barriers and facilitators for implementation of a panel management approach for cardiovascular disease management – appropriate intervention**

|  | **Appropriate interventions** | |
| --- | --- | --- |
| **Domain and Construct** | **Potential Barriers** | **Potential Facilitators** |
| **Intervention characteristics** |  |  |
| Evidence strength & quality |  | Lifestyle is the future centred on self-management, autonomy and care optimization. |
| Adaptability |  | Consult preparatory questionnaires should be tailored to personal characteristics |
| Complexity | Implementation of extra decision support in HIS | Operating manual as simple as possible |
|  | Interventions must be able to be integrated into multiple systems | Access to a technical helpdesk |
|  |  | Intuitive user-interface |
| Design quality and packaging | No insight into hospital data and no direct link to EMR, and therefore multiple systems to view patients' data | Training and education for end-users |
|  | No possibility of bringing your own device | Package of implementation supplies that any practice can use |
| Costs |  | Willingness of patients to purchase blood pressure monitors themselves |
| Responsibility |  | Patients responsible for their own health (data) |
|  |  | Healthcare groups must take responsibility for implementation and scale-up |
| Requirements |  | Caregiver not responsible for technical support |
|  |  | Continuity of reimbursement |
|  |  | Proper education and information to patients |
| Outcomes |  | Consult preparatory questionnaires provide room for conversation during consults and help to stratify patients better |
|  | Lots of data to check, technical problems, distributing the Box and proactively calling patients can give the practice nurse extra work. | The Box supports lifestyle, health awareness, self-management and diagnostics. Resource allocation through fewer consultations. |
| Appropriate intervention identification | Impossibility of using your own device | Interventions are valuable for getting and keeping a better focus on patients |
| **Outer setting** |  |  |
| Patient needs & resources | Worried well' better reached | Increasing degree of self-management, including among the older population |
|  | Proportion of patients need human contact | Different levels of guidance to suit different populations |
|  | Patients freedom of choice must be respected | Offers reassurance to patients |
| Cosmopolitanism |  | Intervention ambassador; implementation manager responsible for the project from the region |
| External policy & incentives | Health insurances continue to urge for lower healthcare costs | Enables health insurance to guarantee continuous adequate care for patients |
| **Inner setting** |  |  |
| Available resources | Limited time healthcare professionals for (technical) support and instructions to the patient | Implementation manager for operational issues |
|  |  | Information meeting for eligible patients |
| Compatibility |  | Connects well to workflow as established infrastructure has been implemented in several practices in the region |
| Access to knowledge and information |  | Platform with frequently asked questions, a newsletter and instruction movies |
|  |  | Clear manual available at the beginning of implementation |
| **Characteristics of individuals** | | |
| Knowledge & beliefs about the intervention | Unsure if the actual change is possible due to patients' own willingness to change | Feedback/reflection possible of data to the patient |
| Self-efficacy | Depends on the patients characteristics (age and intelligence may play a role) |  |
| Individual stage of change | Education level difference | Clear rules surrounding urgency for assistance |
|  |  | Program efficiency |
|  |  | Increase patients' own health awareness |

**Table S4 Potential barriers and facilitators for implementation of a panel management approach for cardiovascular disease management – surveillance of care gaps**

|  | **Surveillance of care gaps** | |
| --- | --- | --- |
| **Domain and Construct** | **Potential Barriers** | **Potential Facilitators** |
| **Intervention characteristics** |  |  |
| Costs | One-off subsidy funds | Resource allocation: more time for other tasks; not necessarily cost-saving |
|  | No compensation for extra hours of implementation | Many patients have their own blood pressure monitor or are willing to buy one |
|  | Cost ahead of benefits |  |
| Outcomes | Not just evaluate the quantitative data, but also qualitative data from professionals | Lowering care burden for healthcare professionals |
|  |  | Important to also include caregiver satisfaction |
| **Process** |  |  |
| Planning | Assessing the incoming data takes (extra) time | Educational training opportunities; clear protocol on how to deal with a particular patient panel |
|  | New ways of working take time and deepening |  |
|  |  | All staff should be informed about the project; proper instruction prior to implementation |
| Formally appointed internal implementation leaders |  | Intervention ambassador; implementation manager responsible for the project in the region |
| Opnion leaders |  | GPs and PNs as faces of the project |
| External change agents |  | External company for the technical issues |
|  |  | Patientfederation for patient interests needs |

Supplementary Table S5. Codebook

| *Topic* | *Short description* |
| --- | --- |
| **I. Current practice** | |
| 1. Workflow | Individuals’ perception of workflows in current  practice and familiarity with facts, truths, and principles related to current practice. |
| 1. Relative advantage | Stakeholders’ perceptions of the advantage of current practice. Including individuals’ positive experiences  with current practice. |
| 1. Disadvantage | Stakeholders’ perceptions of the disadvantage of  current practice. Including individuals’ negative experiences with current practice. |
| **II. Intervention characteristics** | |
| 1. Evidence strength & quality | Stakeholders’ perceptions of the quality and validity of evidence supporting the belief that the intervention  will have desired outcomes. |
| 1. Relative advantage | Stakeholders’ perceptions of the advantage of implementing the intervention versus an alternative solution. Stakeholders’ perception of possible  positive experiences with the intervention. |
| 1. Disadvantage | Stakeholders’ perceptions of the disadvantage of implementing the intervention. Stakeholders’  perception of possible negative experiences with the intervention. |
| 1. Adaptability | The degree to which an intervention can be adapted, tailored, refined, or reinvented to meet local needs. |
| 1. Trialability | The ability to test the intervention on a small scale in the organization, and to be able to reverse course  (undo implementation) if warranted. |
| 1. Complexity | Perceived difficulty of implementation, reflected by  duration, scope, radicalness, disruptiveness, |

|  | centrality, and intricacy and number of steps required  to implement. |
| --- | --- |
| 1. Design quality and   packaging | Perceived excellence in how the intervention is  bundled, presented and assembled. |
| 1. Costs | Stakeholders’ perceptions of the costs of the intervention and costs associated with implementing that intervention including investment, supply and  opportunity costs. |
| 1. Responsibility | Agreements on the responsibility concerning the intervention. |
| 1. Requirements | Stakeholders’ perceptions of the requirements the intervention must meet. |
| 1. Outcomes | Stakeholders’ expectations on outcomes that will potentially be accomplished after implementation of the intervention. Stakeholders’ perception on desired  outcomes accomplished by the intervention. |
| 1. Panel identification | Appropriate empanelment strategy |
| 1. Appropriate intervention | Suggested appropriate interventions linked to panels |
| **III. Outer setting** | |
| A. Patient needs & resources | The extent to which patient needs, as well as barriers  and facilitators to meet those needs are accurately known and prioritized by the organization. |
| B. Cosmopolitanism | The degree to which an organization is networked with other external organizations. |
| **IV. Inner setting** | |

| E. Readiness for implementation | Tangible and immediate indicators of organizational  commitment to its decision to implement an intervention. |
| --- | --- |
| 2. Available resources | The level of resources dedicated for implementation of on-going operation including money, training,  education, physical space, and time. |
| 3. Access to knowledge and information | Ease of access to digestible information and  knowledge about the intervention and how to incorporate it into work tasks. |
| **V. Characteristics of individuals** | |
| A. Knowledge & Beliefs about the intervention | Individuals’ attitudes toward and value placed on the intervention as well as familiarity with facts, truths,  and principles related to the intervention. |
| B. Self-efficacy | Individual belief in their own capabilities to execute  courses of action to achieve implementation goals. |
| C. Individual stage of change | Characterization of the phase an individuals is in, as  he or she progresses toward skilled enthusiastic, and sustained use of the intervention. |
| **VI. Process** | |

| A. Planning | The degree to which a scheme or method of behavior and tasks for implementing an intervention are developed in advance and the quality of those  schemes or methods. |
| --- | --- |
| B. Engaging | Attracting and involving appropriate individuals in implementing and using the intervention through a combined strategy of social marketing,  education, role modeling, training, and other similar activities. |
| 1. Opinion leaders | Individuals in an organization who have formal or informal influence on the attitudes and beliefs of their  colleagues with respect to implementing the intervention. |
| 2. Formally appointed internal implementation  leaders | Individuals from within the organization who have been formally appointed with responsibility for implementing an intervention as coordinator, project  manager, team leader, or other similar role. |
| 4. External change agents | Individuals who are affiliated with an outside entity  who formally influence or facilitate intervention decision in a desirable direction. |

**Supplementary Figure S1. Connect@Heart Panel Management Approach**


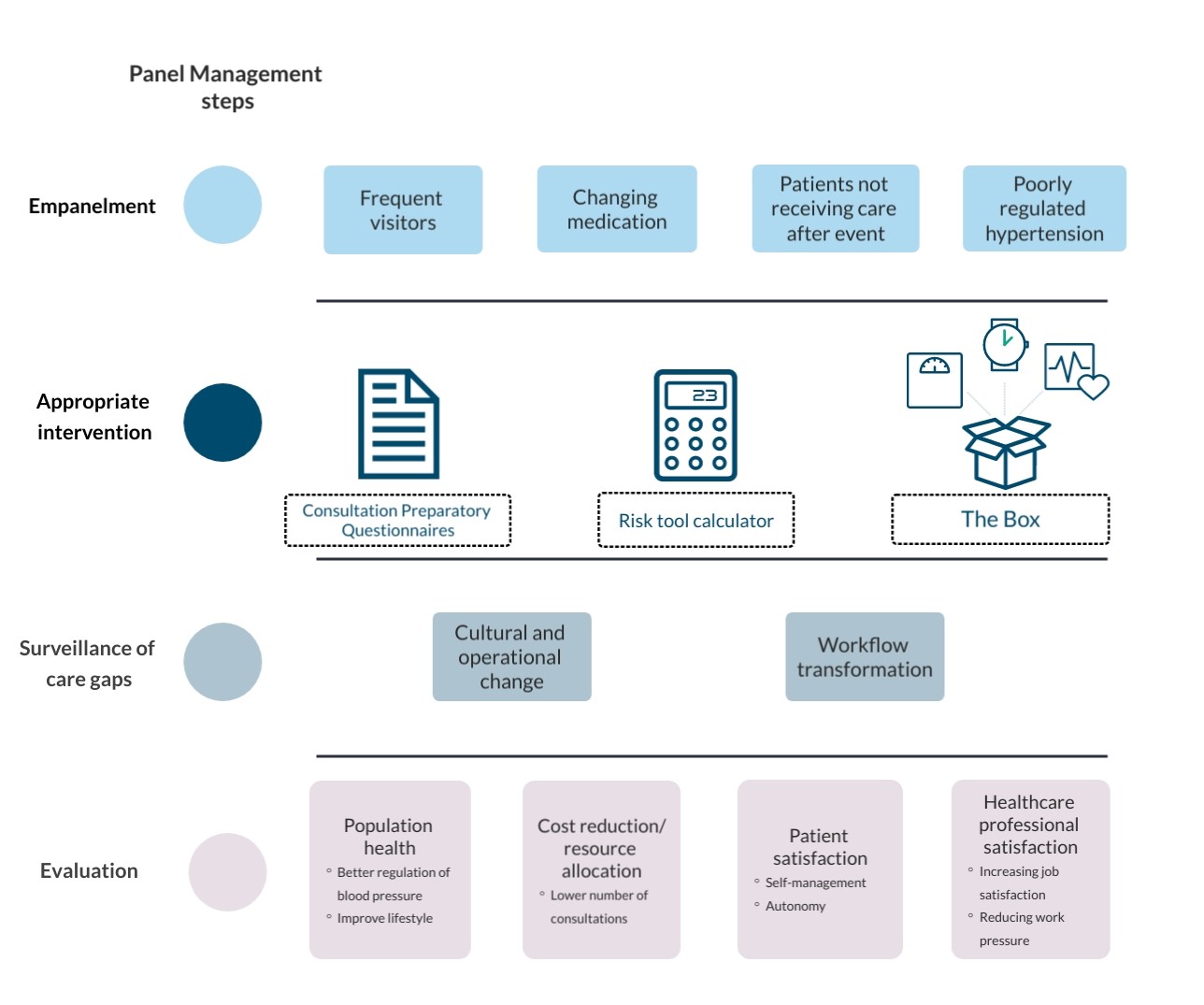
 **Figure S1**

**Empanelment**

**Frequent visitors**

The top three per cent of contacts with a GP account for 15 per cent of all consultations [2]. These individuals typically have more than 10 to 15 consultations per year, with a significant proportion presenting with cardiovascular complaints. In fact, patients with cardiovascular risk management (CVRM)-related issues may have more than 30 consultations per year [3].

**Identifying patients not in chain care after event**

Patients who experience a cardiovascular event may fall into a treatment gap, where they do not receive appropriate CVRM care from their GP or hospital. To address this issue, support is needed to help GPs identify and manage these patients who may "fall between the cracks." This approach can help reduce the risk of recurrent cardiovascular events and related complications.

**Poorly regulated hypertension**

The prevalence of poorly controlled hypertension is noteworthy. Studies conducted in England have demonstrated that only 53% of patients treated for hypertension had a regulated blood pressure of <140/90mmHg [4]. Similarly, a cross-sectional analysis of the Framingham Heart Study revealed that less than half of treated participants (48%) achieved the recommended blood pressure target of <140/90mmHg, with a particularly low rate of achievement (<40%) among those over 75 years of age [5]. Additionally, Dutch research has demonstrated that only 44% of patients with type 2 diabetes achieved the recommended target blood pressure level [6].

**Changing medication**

Antihypertensive therapy typically involves ACE/ARB inhibitors, calcium antagonists, beta-blockers, and diuretics. However, the effectiveness of these agents can vary depending on individual patient characteristics, which may require switching or combining antihypertensive agents [7]. Even though current guidelines are well-supported by evidence from randomized controlled trials, the current protocols for antihypertensive therapy are not highly individualized [8].

**Appropriate interventions**

**The Box**

Previous studies have demonstrated that home blood pressure measurement and monitoring can improve the accuracy of blood pressure measurements and reduce blood pressure levels in patients with difficult-to-regulate hypertension [9], [10]. The Box comprises a blood pressure monitor, scale, and activity meter for home monitoring. Through implementing the Box in primary care, we aim to investigate its potential in patients with increased cardiovascular risk, to empower patients to take control of their health and promote awareness of the importance of a healthy lifestyle.

**Consultation Preparatory Questionnaires**

During a consultation, healthcare providers typically spend significant time asking standard questions to gather information about the patient's health status. While these questions are necessary, they can detract from the opportunity to have meaningful conversations with patients. The Consult Preliminary Questionnaire module aims to alleviate the registration burden for healthcare providers and create more time and attention for patient-centered care.

References

[1] E. (Estee) B. Neuwirth, J. A. Schmittdiel, K. Tallman, and J. Bellows, “Understanding Panel Management: A Comparative Study of an Emerging Approach to Population Care,” *Perm. J.*, vol. 11, no. 3, p. 12, Jul. 2007, doi: 10.7812/TPP/07-040.

[2] R. D. Neal, P. L. Heywood, S. Morley, A. D. Clayden, and A. C. Dowell, “Frequency of patients’ consulting in general practice and workload generated by frequent attenders: comparisons between practices.,” *Br. J. Gen. Pract.*, vol. 48, no. 426, p. 895, 1998, Accessed: Jan. 19, 2022. [Online]. Available: /pmc/articles/PMC1409909/?report=abstract.

[3] K. Kivelä, S. Elo, and M. Kääriäinen, “Frequent attenders in primary health care: A concept analysis,” *Int. J. Nurs. Stud.*, vol. 86, pp. 115–124, Oct. 2018, doi: 10.1016/J.IJNURSTU.2018.06.003.

[4] I. Hajjar and T. A. Kotchen, “Trends in Prevalence, Awareness, Treatment, and Control of Hypertension in the United States, 1988-2000,” *JAMA*, vol. 290, no. 2, pp. 199–206, Jul. 2003, doi: 10.1001/JAMA.290.2.199.

[5] D. M. Lloyd-Jones, J. C. Evans, M. G. Larson, C. J. O’Donnell, E. J. Roccella, and D. Levy, “Differential control of systolic and diastolic blood pressure : factors associated with lack of blood pressure control in the community,” *Hypertens. (Dallas, Tex. 1979)*, vol. 36, no. 4, pp. 594–599, 2000, doi: 10.1161/01.HYP.36.4.594.

[6] A. van der Horst-Schrivers, “Matige bloeddrukregulatie bij patiënten met diabetes mellitus type 2 in de eerste en tweede lijn,” 2004.

[7] D. Bertsimas, A. Rose Ann Borenstein, A. Dauvin, A. Orfanoudaki, and C. Agni Orfanoudaki, “Ensemble machine learning for personalized antihypertensive treatment,” *Nav. Res. Logist.*, Dec. 2021, doi: 10.1002/NAV.22040.

[8] J. B. Byrd, “Personalized medicine and treatment approaches in hypertension: current perspectives,” *Integr. Blood Press. Control*, vol. 9, pp. 59–67, Apr. 2016, doi: 10.2147/IBPC.S74320.

[9] R. J. McManus *et al.*, “Efficacy of self-monitored blood pressure, with or without telemonitoring, for titration of antihypertensive medication (TASMINH4): an unmasked randomized controlled trial,” *Lancet (London, England)*, vol. 391, no. 10124, pp. 949–959, Mar. 2018, doi: 10.1016/S0140-6736(18)30309-X.

[10] J. Hodgkinson *et al.*, “Relative effectiveness of clinic and home blood pressure monitoring compared with ambulatory blood pressure monitoring in diagnosis of hypertension: systematic review,” *BMJ*, vol. 342, no. 7814, Jul. 2011, doi: 10.1136/BMJ.D3621.

**Supplementary Text S2a: topic list interviews with GPs and POHs**

1. **Introduction**
   Welcome and thank you for your participation.
2. **Permission to record, anonymity**
   Prior to proceeding, may I kindly request your permission to record this conversation? The recording is necessary for us to accurately transcribe the interview. Rest assured that your name will not be recorded, and each participant will be assigned a unique code. Your personal information will not be retained in any way.
3. **Purpose**
   As mentioned in the email and accompanying documents, our research team is developing a RPM program with a panel management approach. Through this interview, our goal is to identify patient subsets that can benefit from interventions to improve elements of the quadruple aim within CVRM. Additionally, we will seek your feedback on implementing this prototype. We plan to initiate a pilot study in general practice during the upcoming summer.
4. **Demographic data participants**

- Would you please complete the enclosed questionnaire about your personal information and information from the general practice where you work?

1. **Questions about CVRM**
   *1. Current situation*

- How have you organized CVRM within your practice? (Sub-question: How do you experience this organization?)

  *2. Room for improvement*
- In your opinion, what could be improved within CVRM care? Sub-question: Why, in your opinion, is it not working now?)
- What is needed for this (Sub-question: Why specifically would you want to improve these aspects? How does this manifest?)
- Do you manage to identify all patients eligible for CVRM? Why yes/no?

1. **Questions regarding selection, intervention and implementation**
   Using the provided handout, we will proceed through the steps of panel management and inquire about patient selection and the interventions that may be associated with them. The following questions will be presented to guide the process.

   *3. Identifying the population*

- What added value do you see in empaneling (systematically identifying patients and assigning them to appropriate interventions, see example in the handout with the Box) in cardiovascular risk management of patients at your GP practice? (For you as a doctor? For the patient?) (Subquestions: How does this manifest itself? Which added value matters most to you?)
  - In which patient panels (step 1 in the handout) do you see an added value of empanelment? Which ones are missing?
  - Do you think Connect@Heart reaches these patients? If not; what are you missing?
- Do you see any patients or patient groups within the CVRM population who could potentially take more ownership? And how would you like/could you identify these?

*4. Quadruple aim indicators*

- Are the quadruple aim goals clear?
- Do the identified panels align with these goals? And do you agree with the outcome measures under the quadruple aim goals?

  *5. Intervention*
- Given the panels, what do you think of the possible interventions we have identified in the handout? Which ones do you see as promising? Which ones could start to help?
  - Consult Preparatory Questionnaires
    - To what extent would a consultation preparatory questionnaire help you give more time and attention to the patient? Will it save time?
  - The Box
    - Do you see an added value in bringing the Box along in promoting health awareness for the patient?
      - Do you see the most added value of the Box for patients who are well-regulated or less well-regulated?
    - Do you see lifestyle education, linked to the Box, as supporting your prevention activities with your patients?
- To what extent do the interventions align with the most important panels you indicated? (Sub-question: How could the system align better?)
- What do you think the potential interventions could achieve?
- How well do you think the intervention will meet your patients' needs? In what ways does the intervention meet their needs? E.g. help with self-management?

*6. Implementation*
*The following questions will be about implementing the particular panel management approach from the figure of the handout.*

- How do you envisage the implementation? What possibilities and opportunities do you see and what will be difficult?
- In your view, what prerequisites or criteria must be satisfied by the interventions before you decide to implement them?
- What are factors that could potentially promote or limit implementation?
- What kind of changes or adjustments do you think you need to make when using the interventions (from the figure in the handout) so that they will work effectively in your setting? Do you think you can make these changes? (Sub-question: why/why not?)
- Do you see any changes in the cost structure within your practice when implementing this innovation? Sub-question: How will the new intervention affect payment or revenue for your organization?)
- How well does the intervention fit with existing work processes in your practice? (Sub-question: What are likely problems or complications that may arise? What actions should be initiated for this?)

**Supplementary Text S2b: topic list focusgroups with GPs, POHs, health insurers, project managers and IT consultants**

*What do you think about panel management of CVRM patients?*

- Does panel management of CVRM patients add value to the implementation of STEVIT?
  - If yes, why? If no, why not?
- Do you see attached panels as appropriate panels and do they add value to the use of the Box and consultation preparatory questionnaires?
- What could such a system add to the care around CVRM?
  - What advantages does the intervention have compared to existing programmes?
  - What disadvantages does the intervention have compared to existing programmes?
- How well does the intervention fit with your setting's existing work processes and practices?
  - What are the likely problems or complications that may arise?
- Are there any components that should be changed in the panel management? If so, which are these?

***How*** *could panel management be implemented in practice?*

- What tools are available to implement the intervention?
  - How do you access these tools?

***What*** *do you think about adding consultation preparatory questionnaires on top of the Box?*

- For which patient groups would this be most valuable?

***Who*** *are needed to implement IMDI?*

- What key stakeholders do you need to get on board for this intervention?
- How well does the intervention align with your setting's existing work processes and practices?
  - What are the likely problems or complications that may arise?
